# Human-in-the-loop Bayesian optimization of wearable device parameters

**DOI:** 10.1371/journal.pone.0184054

**Published:** 2017-09-19

**Authors:** Myunghee Kim, Ye Ding, Philippe Malcolm, Jozefien Speeckaert, Christoper J. Siviy, Conor J. Walsh, Scott Kuindersma

**Affiliations:** 1 John A. Paulson School of Engineering and Applied Sciences, Harvard University, Cambridge, MA, United States of America; 2 Wyss Institute for Biologically Inspired Engineering, Harvard University, Cambridge, MA, United States of America; 3 Department of Biomechanics and Center for Research in Human Movement Variability, University of Nebraska Omaha, Omaha, NE, United States of America; Chongqing University, CHINA

## Abstract

The increasing capabilities of exoskeletons and powered prosthetics for walking assistance have paved the way for more sophisticated and individualized control strategies. In response to this opportunity, recent work on *human-in-the-loop* optimization has considered the problem of automatically tuning control parameters based on realtime physiological measurements. However, the common use of metabolic cost as a performance metric creates significant experimental challenges due to its long measurement times and low signal-to-noise ratio. We evaluate the use of Bayesian optimization—a family of sample-efficient, noise-tolerant, and global optimization methods—for quickly identifying near-optimal control parameters. To manage experimental complexity and provide comparisons against related work, we consider the task of minimizing metabolic cost by optimizing walking step frequencies in unaided human subjects. Compared to an existing approach based on gradient descent, Bayesian optimization identified a near-optimal step frequency with a faster time to convergence (12 minutes, *p* < 0.01), smaller inter-subject variability in convergence time (± 2 minutes, *p* < 0.01), and lower overall energy expenditure (*p* < 0.01).

## 1 Introduction

Recent advances in wearable assistive devices have demonstrated great potential for improving the metabolic economy during walking [1–12]. The behavior of these systems is typically governed by a set of control parameters prescribing, for example, the timing and magnitude of assistive forces being applied to a joint [1–9]. Typically, these parameters are set using measurements from biomechanics on an average population [1, 6–8, 13]. However, the data collected during these studies have also shown significant inter-subject variability in response to any given assistive strategy, suggesting that device parameters that help one subject may *hinder* another [14]. This leads naturally to the hypothesis that overall performance across subjects could be significantly improved by tuning control parameters for each individual.

Subject-specific adjustment of assistance profiles is conventionally performed using expert knowledge and observation [3, 15]. However, with increased control parameter dimensionality in wearable devices [4, 7, 16, 17] and a growing population of users, manual tuning becomes impractical [18]. Human-in-the-loop (HIL) optimization methods instead attempt to automatically identify low-cost parameter values by using real-time physiological signals such as metabolic cost, removing the need for expert intervention or an exhaustive search [11, 19, 20].

Metabolic cost is typically inferred indirectly by averaging noisy respiratory rate measurements over a number of breaths. Using this approach, a minimum of 1–2 minutes of breath measurements must be gathered to estimate metabolic cost (assuming a breath rate between 0.2 and 0.3 breaths per second) [21–23]. In addition, conventional experimental procedures demand a warm-up period due to slow and nonlinear mitochondrial dynamics [24] and long transit time [25]. The combination of these two factors requires 4–6 minutes walking for each condition, which is challenging for experiments that include several different walking conditions or for individuals with impairments (e.g., active individuals with amputation [14] or subjects with pathological gaits [26]). In addition, increased physical exertion due to long-duration experiments may result in cardiopulmonary drift and subject discomfort [27], which can affect the accuracy of metabolic cost measurement.

There are two complementary ways to reduce the overall experimental time in HIL studies: reducing metabolic estimation time (i.e. time required per sample) and reducing the number of sample conditions. Recent work has developed a metabolic cost estimation method that requires fewer breaths at the cost of some accuracy [25], but this method still requires 90 seconds on average for each measurement. In addition, tuning the estimator parameters requires tens of conditions, further increasing subject exertion. This paper evaluates the potential of Bayesian optimization [28–33], a sample-efficient and noise-tolerant global optimization method, to significantly reduce experimental times through parsimonious evaluation of walking conditions.

Several HIL optimization methods have been explored previously, including response surface methods [19], gradient descent [19], and evolutionary algorithms [11]. These methods have been explored as an alternative to discrete grid search (i.e. parameter sweeping), which evaluates a fixed number of conditions (often based on pilot study results), then selects the condition with lowest average cost. Thanks to the curse of dimensionality, the number of samples required to cover the parameter space at discrete intervals increases exponentially with the number of parameters. Response surface methods [19] use generalization to relieve some of this sampling burden by fitting a parametric function (e.g., a polynomial) to a set of sample points and using the resulting surface to approximate the cost as a function of the control parameters. In many cases, it can be difficult to know the parametric mapping between control parameters to metabolic cost a priori, so these methods can be prone to overfitting and bias [34, 35].

Felt et al. showed that gradient descent can efficiently perform HIL metabolic cost optimization in an unassisted step frequency optimization study [19]. Their experiment was designed to mimic the essential attributes of assistive device control optimization without an assistive device. In a later study, the optimization method was used to find a near-optimal parameter of a wearable device faster than discrete grid search [20]. Although stochastic gradient descent algorithms are guaranteed to find local minima under mild assumptions, their sample complexity depends strongly on the signal-to-noise ratio of the cost [36]. Evolutionary algorithms based on covariance matrix adaptation (CMA-ES) have also been considered [11, 37]. CMA-ES does not directly estimate the derivative, but instead can be loosely thought of a sampling-based 2nd-order optimization method. However, each iteration requires multiple parameter evaluations [38], leaving open the possibility for sample efficiency gains.

Bayesian optimization methods generalize response surface methods using nonparametric regression models and principled metrics for selecting new data points [35, 39–41]. Given initial measurements, Bayesian optimization optimizes a posterior distribution of metabolic cost over the control parameter space. The posterior distribution is represented as Gaussian process where prior knowledge about signal noise and surface shape can be easily incorporated (e.g., heart rate [42]). Given this posterior distribution, a variety of parameter selection criteria can be computed that incorporate knowledge of low-cost points as well as regions of high uncertainty (e.g., where no samples yet exist).

We designed a step frequency optimization experiment, based on Felt et al. [19], to demonstrate the feasibility and efficacy of Bayesian optimization. In addition to excluding confounding effects from wearable devices—such as low-level parameter tracking performance—the step frequency experiment design also allows for direct comparison against previously published results. We hypothesized that Bayesian optimization will require fewer parameter evaluations to recover low-cost step frequencies compared to gradient descent and result in similar or better metabolic reduction (depending on the prevalence of local minima). We also hypothesized that the fast convergence of Bayesian optimization will result in overall lower subject energy expenditure during the experiment as compared to the gradient descent. We expected the results of this study to inform future work on efficient optimization of individualized control strategies in wearable devices.

## 2 Methods

An experiment was designed whereby a subject’s step frequency was prescribed using a metronome and their metabolic response was estimated using breath measurements. The study included three experimental conditions: discrete grid search, gradient descent [19], and Bayesian optimization. Each condition was presented on a separate day and each of the three methods was performed for a fixed number of trials. The quality of the solution returned by both gradient descent and Bayesian optimization were evaluated by comparing against the estimated optimal step frequencies from the discrete grid search. The convergence times for both methods were evaluated offline using several criteria.

### 2.1 Experimental protocol

#### 2.1.1 Participants

Nine healthy participants (five male and four female; age, 27.1 ± 4.8 years; mass 65.8 ± 9.7 kg; height, 173.2 ± 8.7 cm; mean ± standard deviation) were recruited for the study. We excluded one subject based on outlier analysis [43] on time to convergence. The subject diverged during the gradient descent condition, perhaps due to fatigue (Respiratory Exchange Rate > 1.5 [44]). All participants provided written informed consent prior to participating. The study was approved by the Harvard Longwood Medical Area Institutional Review Board on Human Studies listed below. HMS IRB number: 22086 Harvard Faculty of Medicine Office of Human Research Administration, 90 Smith Street, 3rd Floor, Boston, MA 02120.

#### 2.1.2 Experimental conditions

Participants experienced one optimization method per day for three days. Discrete search was performed on the first day while the gradient descent and Bayesian optimization methods were randomly assigned to the second and third day to mitigate potential order effects. Each condition involved walking for approximately 60 minutes and testing days were separated by at least 48 hours to avoid fatigue effects. All walking bouts were on a treadmill (Sole fitness TT8 Treadmill, SOLE Fitness, USA) at 1.25 *m* ⋅ *s*^−1^ while equipped with a respiratory device to record their metabolic cost for all the standing and walking bouts.

At the beginning of the first day, resting metabolic cost was measured during quiet standing for six minutes. A ten minute warm up period was then conducted and subjects were asked to “walk normally,” unguided by the metronome. During this period, the subject’s preferred step frequency was measured. Subjects had a three minute break after the warm up. Subsequently, they underwent nine six minute walking bouts following the metronome at nine different step frequencies: their preferred step frequency and 25%, 15%, 10%, and 5% below and above their preferred step frequency. These step frequencies were chosen to cover the entire parameter space while preventing subject fatigue by removing two conditions compared to Felt et al. [19]. The sequence of the nine walking bouts was randomized to minimize any fatigue, order, and learning effects. Adequate rest of on average two minutes was given between walking bouts to allow physical recovery. On the second and third days, we conducted the same six minutes standing and ten minutes warm up periods before optimization.

The gradient descent condition was composed of three walking bouts, after the standing and warm up periods. Each walking bout was approximately 15 minutes and consisted of 5 gradient descent iterations (total of 15 iterations). For each iteration, the subject walked for a period of 30 breaths at 5% below and 5% above the step frequency commanded by the gradient descent (total of 60 breaths), which took approximately 3 minutes total per iteration, depending on each subject’s breathing rate. Subjects were given 5 minutes of rest between walking bouts.

The Bayesian optimization condition was composed of two walking bouts after the standing and warm up periods. Each walking bout was approximately 20 minutes and consisted of 10 Bayesian optimization iterations including 3 initial exploration points. For each iteration, the subject walked for a period of 40 breaths at the step frequency commanded by the Bayesian optimization, which typically took 2 minutes on average. Subjects were given 5 minutes of rest between walking bouts.

#### 2.1.3 Data collection

Measured step frequency, commanded step frequency, and metabolic cost were collected throughout all of the conditions. For the gradient descent and Bayesian optimization conditions, all final and intermediate algorithmic parameters were collected.

### 2.2 Human-in-the-loop experimental system

**Fig 1:**
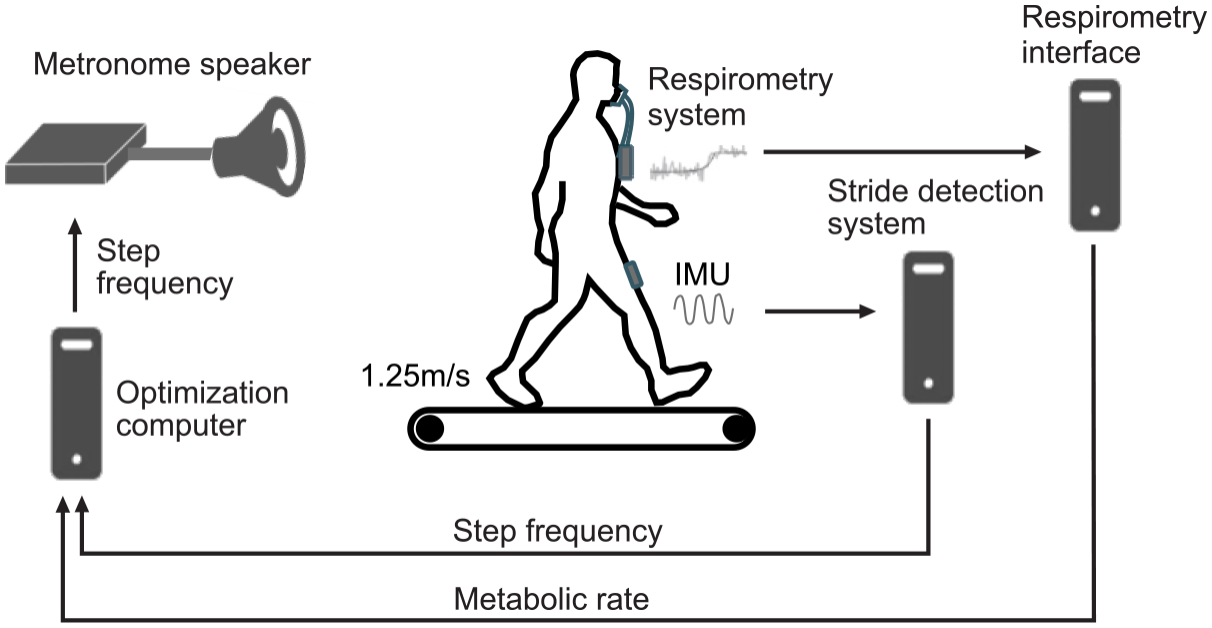
Experimental system. Our experimental system is composed of (1) a metabolic cost estimation system, (2) a step detection system, (3) an optimization computer, and (4) a metronome.

We configured the HIL experimental system as shown in Fig 1. It consisted of (1) a respiratory device (K4b2, Cosmed, Roma, Italy) and an interface computer for measuring metabolic cost, (2) an IMU sensor (VN-100 Rugged IMU, Vectornav Technologies, USA) and a step detection computer for step frequency estimation, (3) an optimization computer, and (4) a metronome speaker. The respiratory device was connected to the respiratory computer through a serial port and communication was done using the software from Felt et al. [19, 45]. The IMU sensor was attached to the anterior part of the subject’s thigh and communications with the stride detection computer was established using a serial connection. All the computers were connected with ethernet cables through a network switch and the Arduino-controlled metronome was directly connected to the optimization computer with a USB cable.

We read the respiratory output at a sampling frequency of 100 Hz through the serial connection while a customized Matlab script converted the carbon dioxide and oxygen rate from the respiratory device into metabolic cost using the Brockway equation [46] on a breath-by-breath basis. Then we estimated the *instantaneous energetic cost* [19], *c*, assuming a first-order dynamic model of the respiratory response [25]. First we assume the energetic cost is a linear function of the measured step frequency,

$$\begin{array}{c} c\left(x\right)=\lambda_{1}x+\lambda_{0}, \end{array}$$ (1)

where *λ*_1_, *λ*_0_ are coefficients and *x* is the measured step frequency. The dynamics of the respiratory response is modeled as

$$\begin{array}{c} r_{i}=\left(1-\frac{h_{i}}{\tau }\right)r_{i-1}+\frac{h_{i}}{\tau }c\left(x\right), \end{array}$$ (2)

where *r*_*i*_ is the respiratory response at breath index *i*, *τ* is a time constant, and *h*_*i*_ is elapsed time since the previous breath [19].

We used a linear model of instantaneous energetic cost for the gradient descent condition and a zero-order model (*λ*_1_ = 0) for Bayesian optimization. The coefficients of the instantaneous energetic cost were calculated by inverting the dynamics Eq (2) and solving a regression problem using the collected respiratory data at each iteration. We used a total of 60 breaths, two sets of 30 breaths for the gradient descent [19]. For the Bayesian optimization, 40 breaths were used. We used a time constant of 42 seconds for both algorithms [25].

Step frequency was inferred from the estimated thigh angle using the IMU. The stride time was calculated as the time between two consecutive maxima of the thigh flexion angle [7]. Stride frequency was taken to be the reciprocal of stride time and it was doubled to compute step frequency assuming symmetry. Following the methods from Felt et al. [19], the average step frequency for the past four steps was calculated as the subject’s measured step frequency.

**Fig 2:**
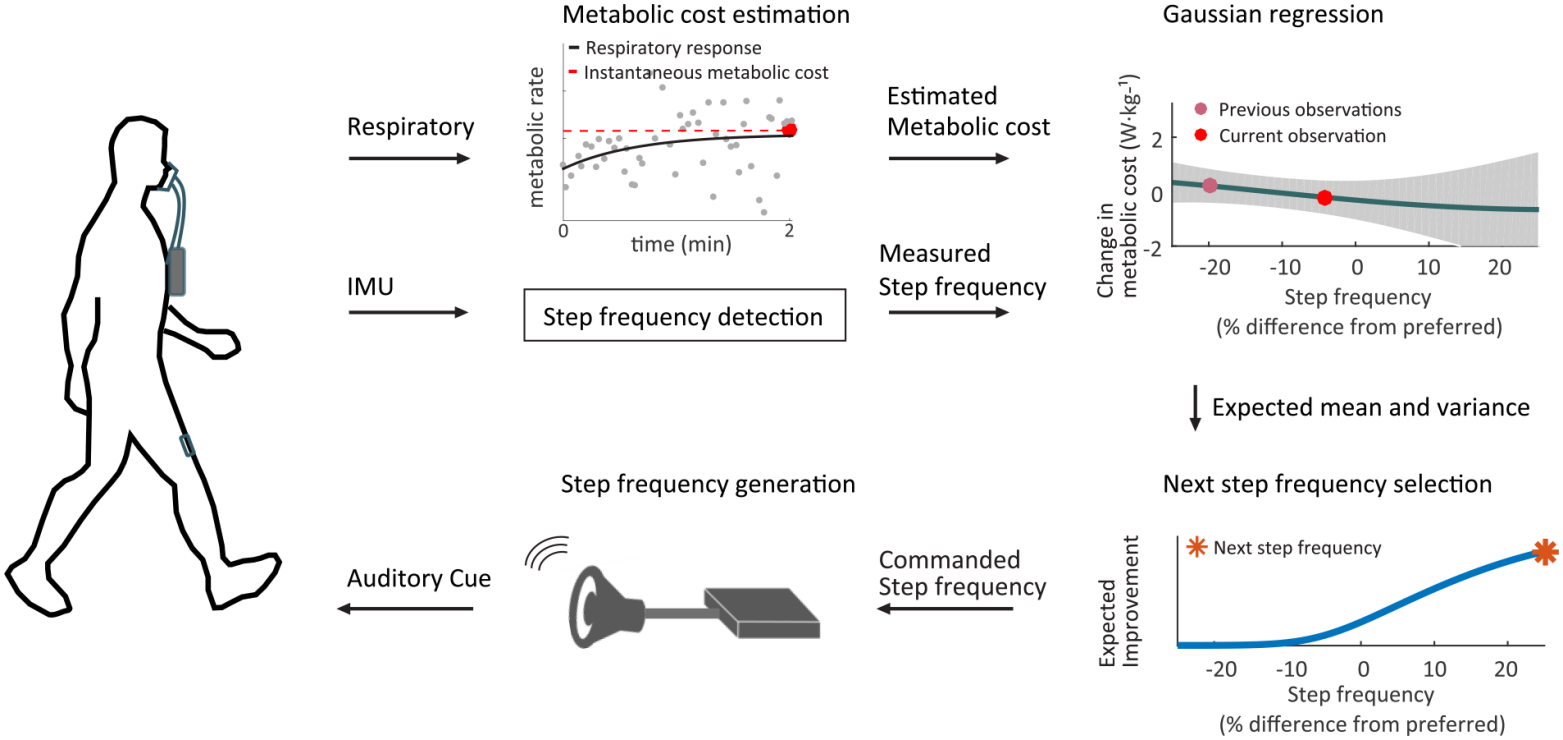
Human-in-the-loop Bayesian optimization. The metabolic cost was estimated using 40 breaths of respiratory data (approximately two minutes altogether) at a step frequency prescribed via metronome. Using the measured step frequency and the estimated metabolic cost, the algorithm computed a posterior distribution of metabolic cost as function of the step frequency. The resulting distribution was used with the expected improvement metric to select the next step frequency.

For all experimental conditions, the estimated instantaneous metabolic cost and measured step frequency were sent to the optimization computer. With the estimated cost and measured step frequency, both algorithms generated the command step frequency at the beginning of each iteration (Figs 1 and 2 for Bayesian optimization). For the discrete grid search condition, the nine different step frequencies were directly commanded by the metronome speaker.

### 2.3 Optimization methods

#### 2.3.1 Gradient descent

The gradient descent method searched for an optimal step frequency by taking steps proportional to the estimated negative gradient of the metabolic cost at the current prescribed step frequency [19]. The algorithm was randomly initialized at either 20% above or 20% below the subject’s preferred step frequency (*x*^*pref*^), then the algorithm ran for 15 iterations.

The gradient of cost function was estimated by sampling at perturbed values, ±*δ*_*n*_, below and above the current step frequency, *x*_*n*_, and computing the symmetric derivative,

$$\begin{array}{c} J_{n}=\frac{c\left(x_{n}+\delta_{n}\right)-c\left(x_{n}-\delta_{n}\right)}{2\delta_{n}}\approx \nabla_{x}c\left(x_{n}\right), \end{array}$$ (3)

where *c*(*x*) is the instantaneousness metabolic cost and *δ*_*n*_ was taken to be 5% of the subject’s preferred step frequency (*δ*_*n*_ = 0.05 ⋅ *x*^*pref*^). The gradient descent algorithm then took small steps proportional to the negative of the gradient of the metabolic cost:

$$\begin{array}{c} x_{n+1}=x_{n}-\alpha_{n}J_{n}, \end{array}$$ (4)

where *α*_*n*_ is a positive scalar that determines the magnitude of the update. This can be a constant chosen a priori or adjusted online as a function of the iteration number, *n*. Following prior work [19], we used the schedule

$$\begin{array}{c} \alpha_{n}=\frac{A_{0}\alpha_{0}}{A_{0}+n^{\gamma }}, \end{array}$$ (5)

where *A*_0_ = 3, *α*_0_ = 4 × 10^−4^ Hz mL^−1^ min^−1^, and *γ* = 1.

#### 2.3.2 Bayesian optimization

Bayesian optimization proceeded by iteratively estimating the posterior distribution of metabolic cost as a function of step frequency (represented as a *Gaussian process* [41]), then globally selecting the next step frequency to evaluate based on *Expected Improvement* (EI) [28, 30]. After each step frequency command, 40 breaths of respiratory data were collected and a new approximated instantaneous metabolic cost value was added to the data set. This processes is illustrated in Fig 2.

**Fitting metabolic distributions using Gaussian process regression**: Gaussian processes can loosely be thought of as a generalization of the multivariate Gaussian distribution to continuous domains (i.e. a distribution over functions) [41]. In the present setting, we aim to represent the distribution of metabolic cost, *c*(*x*), as a function of the continuous step frequency parameter, *x*, using a small number of samples obtained during human subject experiments. The prior of a Gaussian process is parameterized by mean, *μ*(*x*), and covariance, *k*(*x*, *x*′), functions. As is standard practice, we set the mean to be zero and the covariance function as the anisotropic squared exponential kernel,

$$\begin{array}{c} k\left(x,x^{\prime }\right)=\sigma^{2}\exp\left(-\frac{1}{2}\left(x-x^{\prime }\right)M\left(x-x^{\prime }\right)\right)+\sigma_{n}^{2} \end{array}$$ (6)

where *σ*^2^ is the signal (metabolic cost) variance, *M* is a diagonal matrix of the length scales, diag(*l*^−2^), that capture the sensitivity of the cost with respect to changes in input parameters, and $\sigma_{n}^{2}$ is the global noise variance. In our case, *M* is a scalar corresponding to the length scale of the single step frequency input. The values of *σ*, *l*, and *σ*_*n*_ are called *hyperparameters* (***θ*** = [*σ*, *l*, *σ*_*n*_]) because they govern the behavior of the model, but are distinct from the parameters (data) used to compute posterior distributions. Rather than selecting hyperparameters a priori, after each iteration they were set to values that maximized the log marginal likelihood of the data, $D=\left\{X,y\right\},X=\left[x_{1},\ldots ,x_{n}\right],y={\left[y_{1},\ldots ,y_{n}\right]}^{T}$ [41]. In our experiments, this optimization was performed using Matlab’s fmincon solver with 10 random initializations to avoid poor local minima.

Samples of the metabolic cost are assumed to have additive independent and identically-distributed (i.i.d.) noise,

$$\begin{array}{c} y\left(x\right)=c\left(x\right)+\varepsilon ,\;\;\;\;\varepsilon ∼N\left(0,\sigma_{n}^{2}\right). \end{array}$$ (7)

Given the Gaussian process prior and data, D, the posterior (predictive) metabolic distribution can be computed for a step frequency, x_*_, as $c\left(x_{*}\right)\equiv c_{*}∼N\left(E\left[c_{*}\right],s_{*}^{2}\right)$,

$$\begin{array}{ccc} E[c_{*}] & = & k_{*}^{T}{\left(K+\sigma_{n}^{2}I\right)}^{-1}y,\\ s_{*}^{2} & = & k\left(x_{*},x_{*}\right)-k_{*}^{T}{\left(K+\sigma_{n}^{2}I\right)}^{-1}k_{*}, \end{array}$$

where k_*_ = [*k*(*x*_1_, x_*_), *k*(*x*_2_, x_*_), …, *k*(*x*_*n*_, x_*_)]^*T*^ and *K* is the positive-definite kernel matrix, [*K*]_*ij*_ = *k*(*x*_*i*_, *x*_*j*_) [41].

**Selecting step frequencies by maximizing expected improvement**: After computing the metabolic posterior given all data, the next step frequency is selected by maximizing expected improvement (EI) [28, 47]. Expected improvement is defined as the expected reduction in cost, or *improvement*, over the the best parameters previously evaluated. The improvement of a parameter x_*_ is defined as

$$\begin{array}{c} I_{*}=\left\{ \begin{array}{cc} \mu_{\text{best}}-c_{*} & \text{if}\;c_{*}<\mu_{\text{best}},\\ 0 & \text{otherwise,} \end{array}\right. \end{array}$$ (8)

where $\mu_{\text{best}}=\min_{i=1,\ldots ,N}E\left[c\left(x_{i}\right)\right]$. Since the predictive distribution is Gaussian, the expected value of I_*_ is

$$\begin{array}{ccc} \text{EI}(x_{*}) & = & \int_{0}^{\infty }I_{*}p\left(I_{*}\right)dI_{*},\\ & = & s_{*}\left(u_{*}\Phi \left(u_{*}\right)+\phi \left(u_{*}\right)\right), \end{array}$$ (9)

where $u_{*}=\left(\mu_{\text{best}}-E\left[c_{*}\right]\right)/s_{*}$, and Φ(⋅) and *ϕ*(⋅) are the CDF and PDF of the normal distribution, respectively. If s_*_ = 0, expected improvement is defined to be 0.

**Fig 3:**
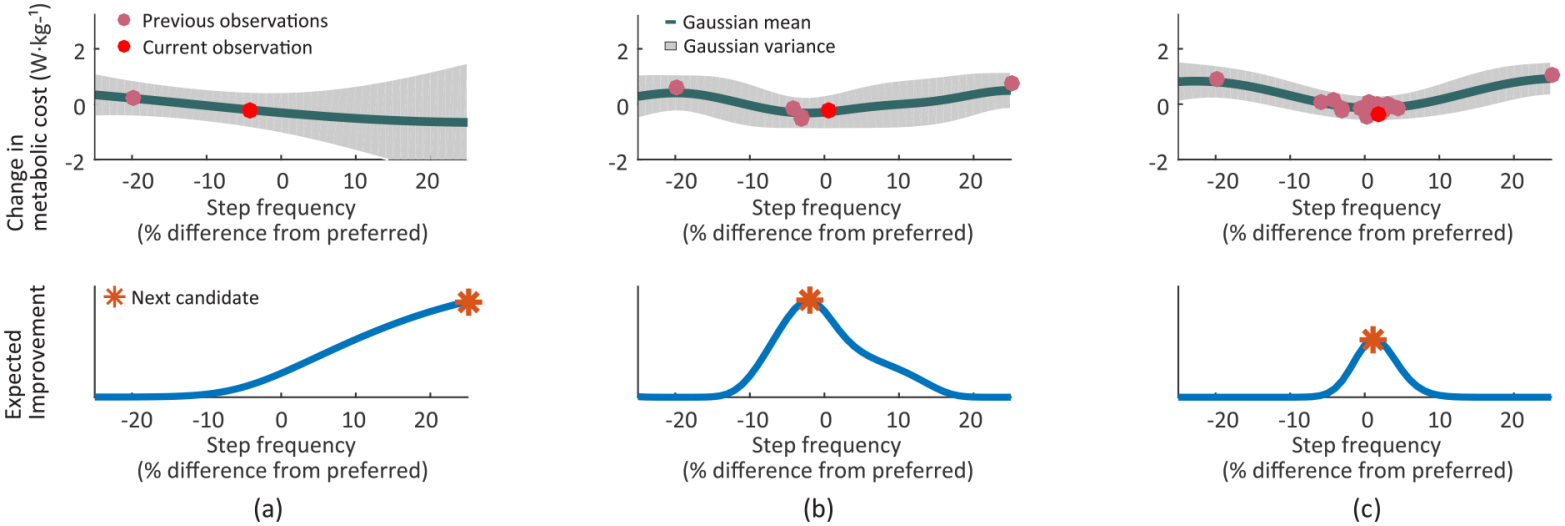
Bayesian optimization process. After collecting two data points, the posterior distribution was calculated (a, top) and expected improvement (EI) was estimated (a, bottom). By maximizing EI, the new candidate point was chosen and the cost was obtained. A new posterior is then computed (b) and the process was repeated until the termination criterion met (c). Above, we used hypothetical data and reduced the exploration points from three to two for a simple explanation.

At each iteration, we maximized expected improvement using Matlab’s fmincon solver with 10 random restarts to avoid poor local optima (Figs 2 and 3 bottom, *).

**Initialization**: Selecting parameters based on distributions fit using very little data can lead to myopic sampling and premature convergence [48]. It is therefore common practice to incorporate a (pseudo-)random sample of *n*_0_ initial parameters prior to performing optimization. In our experiments, the metabolic cost was measured at three initial step frequencies randomly selected from intervals of 75 to 91.7%, 91.7 to 108.3%, and 108.3 to 125% of preferred step frequency, respectively. After this initial exploration, the optimization was performed for 20 iterations by iteratively (1) optimizing the model hyperparameters given the data and (2) maximizing EI to select the next step frequency.

### 2.4 Data analysis

#### 2.4.1 Metabolic landscape baseline

Since the true energetically optimal step frequency was not known a priori, we estimated the metabolic landscape using discrete grid search to evaluate the quality of the optimization results. The metabolic cost was averaged during the last two minutes of each step frequency condition, the standing metabolic cost was subtracted, and the resulting value was normalized by body mass. We fit a Gaussian process model to the data and considered the step frequency with the minimum mean metabolic cost to be our baseline minimum energy solution. To facilitate comparison with prior studies [19], we also used the subject’s preferred step frequency as a secondary baseline.

#### 2.4.2 Time to convergence

In post-hoc analyses, we compared the time to convergence of gradient descent and Bayesian optimization by applying two different termination criteria to the data collected in our experiments. The first criterion terminated when the iteration-to-iteration change in step frequency fell below a termination threshold, *ϵ*_*sf*_, while the second terminated when the change in metabolic cost fell below a threshold, *ϵ*_*mc*_ [33, 49]. Using these two criteria, we determined the time to convergence for each method and the corresponding error in the step frequency at convergence.

The threshold values were set programmatically using a separate pilot dataset. Briefly, we iteratively lowered the threshold, choosing the highest value that resulted in a step frequency at termination within 10% of the true preferred step frequency, as determined by a discrete grid search. These conditions were met when *ϵ*_*sf*_ = 0.41% and *ϵ*_*mc*_ = 2% for gradient descent, and *ϵ*_*sf*_ = 1% and *ϵ*_*mc*_ = 2% for Bayesian optimization. These values represent a reasonable trade-off between time to termination and error at termination. For example, instead using *ϵ*_*sf*_ = 1% in a gradient descent simulation resulted in fast convergence (12 minutes on average) at the cost of high error (12% on average), while smaller threshold values increased time to termination dramatically with only small decreases in error.

#### 2.4.3 Energy expenditure estimation during optimization

**Fig 4:**
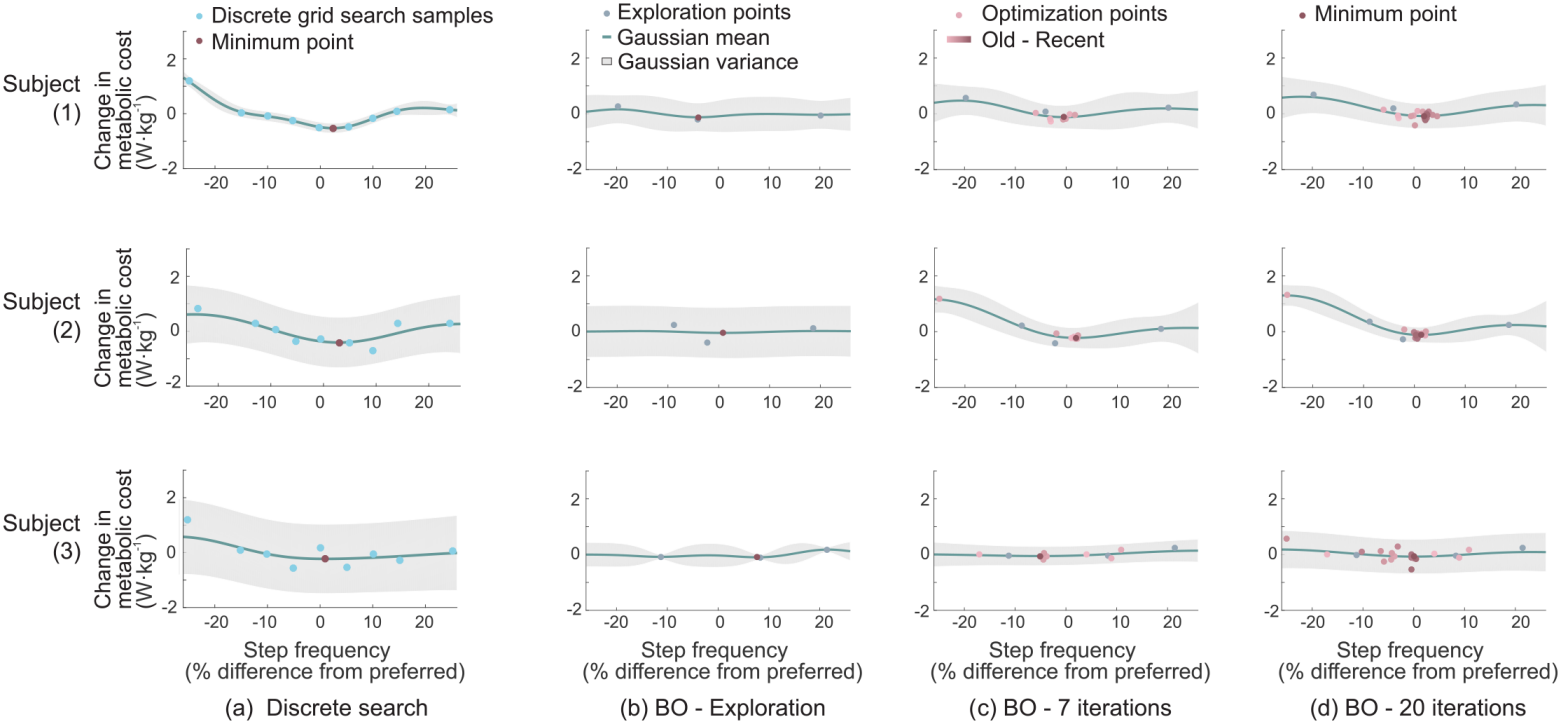
Gaussian process fits to discrete grid search results (a) and Bayesian optimization (BO) results at three time points in our study (b, c, d) for three subjects (subjects 1, 2, 3). The *y* axis shows normalized metabolic cost calculated by subtracting normalized mean metabolic cost. Subfigure (b, c, d) show posterior distributions after three initial exploration step frequencies (a), after seven iterations before the experimental break, and the final posterior distribution after 20 iterations (d). In each case, Bayesian optimization quickly identified a low-cost step frequency within 10 iterations despite qualitative differences in the cost landscape.

We estimated energy expenditure during the optimization process by integrating the constant metabolic cost for each step frequency over time. The metabolic landscape was obtained from the Gaussian process of the discrete grid search results (Fig 4(a)) to minimize day-to-day variability.

#### 2.4.4 Statistical analysis

The significance of observation was evaluated using statistical analysis. Considering the number of participants (n = 9), we first conducted the Jarque-Bera normality test [50]. Considering the low p-value from the test (*p* > 0.1), we conducted Wilcoxon signed-ranks test [51], an alternative non-parametric test to the paired t-test, on time to convergence, error rate, and energy expenditure at significance level 0.05. We also ran Levene’s test for Equality of Variances [52] to examine inter-subject variability on time to convergence for both optimization methods at significance level 0.05.

## 3 Results

**Fig 5:**
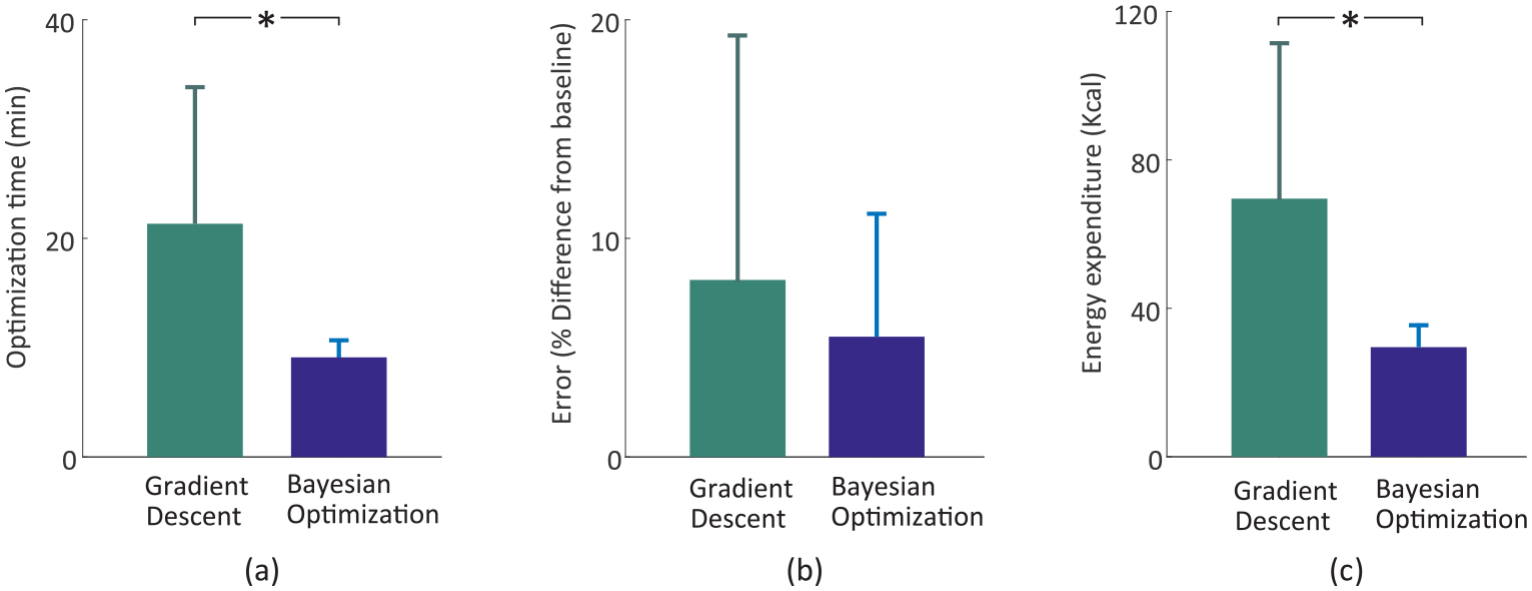
Optimization time, absolute error, and expected energy for instantaneous cost gradient descent and Bayesian optimization. (a) Bayesian optimization found an optimal point in a shorter time (*, *p* < 0.01). The smaller standard deviation for Bayesian optimization shows that inter-subject variability was also smaller (*p* < 0.01). (b) We observed a lower average error at convergence for Bayesian optimization, although this difference was not statistically significant. (c) The total estimated energy expenditure during optimization was significantly lower for Bayesian optimization (*, *p* < 0.01).

While the instantaneous cost gradient search required seven iterations (14 step frequency samples) to converge, corresponding to approximately 21 minutes of experimental time, Bayesian Optimization found a near-optimal parameter within five iterations, or approximately 10 minutes of experimental time (Table 1, Fig 5(a), *p* < 0.01 for both termination criteria). The shorter average time to convergence in Bayesian optimization resulted in lower estimated energy consumption (69.6 ± 41.9 Kcal for the instantaneous cost gradient descent vs. 31.5 ± 6.5 Kcal for Bayesian optimization, *p* = 0.004) with small inter-subject variability (*p* = 2 ⋅ 10^−5^) compared to gradient descent (Fig 5(c)).

**Table 1:** Performance of Bayesian optimization and gradient descent: number iterations and error at convergence compared to a baseline.

	Bayesian optimization		Gradient descent	
Convergence criterion	Step frequency	Metabolic cost	Step frequency	Metabolic cost
Number of iterations	4.9 ± 0.9	5.2 ± 1.1	7.1 ± 4.1	6.4 ± 2.1
% Error (vs. sweep fit)	5.5 ± 4.1	5.4 ± 4.0	8.1 ± 7.3	9.3 ± 6.3
% Error (vs. preferred)	7.0 ± 5.1	7.0 ± 5.4	8.6 ± 5.1	9.2 ± 3.3

Variability in time to convergence between subjects was also lower for Bayesian optimization, as shown by the smaller standard deviation in Fig 5(a) (*p* < 0.01 for both termination criteria). We observed that Bayesian optimization had a lower average error—defined as difference between optimized step frequency at convergence and the minimizing step frequency from discrete grid search—although this difference was not statistically significant (Table 1, Fig 5(b)).

## 4 Discussion

We performed a HIL optimization experiment using Bayesian optimization to automatically identify step frequencies that minimize metabolic cost. The efficient global parameter selection method of Bayesian optimization led to faster time to convergence and lower metabolic energy expenditure from the participants with smaller error as compared to gradient descent. These results suggest that Bayesian optimization is a promising method for HIL optimization research.

Bayesian optimization quickly and reliably found a near-optimal step frequency, even when subjects exhibited high noise (as in Fig 4). After running both algorithms for a fixed number of iterations, we evaluated two convergence criteria based on changes in the commanded step frequency and metabolic cost. In both cases, Bayesian optimization showed faster convergence than instantaneous cost gradient descent across all subjects (Table 1, Fig 5(a)). The accuracy of Bayesian optimization was also improved compared to gradient descent (Fig 5(b)). Applying additional convergence criteria did not change the trend of fast and accurate optimization (e.g., hyperparameters). These characteristics demonstrate the ability of Bayesian optimization to efficiently search for the optimal step frequency during short experiments with noisy measurements of metabolic cost. This fast convergence led to a lower total metabolic expenditure, as calculated by integrating estimated energy expenditure over time to convergence, making these methods attractive for patients with smaller energy budgets.

One potential limitation of the current Bayesian optimization approach is the assumption of stationarity of the metabolic landscape. The relationship between wearable device assistance and metabolic cost can be time-varying. For example, it is possible that metabolic cost in a specific walking condition decreases due to wearer’s adaptation, or increases due to changes in body temperature or cardiovascular drift [53, 54]. Gradient descent algorithms [19, 20] and other local search methods [11] are less sensitive to those effects because they use only recent data to select subsequent parameter values. As implemented, our Bayesian optimization approach uses all data collected in previous iterations. We evaluated a cross validation method [55] that assigned higher weight to recent data [35, 56], but we found that underweighting early samples led to re-exploration and longer experimental times. This limitation of Bayesian optimization could be partially addressed through careful experimental protocol design to minimize variation on the steady state metabolic cost, such as completing a training for adaptation is completed before the start of the optimization protocol begins [4, 57–59] and minimizing fatigue by designing a short protocol with sufficient rest or using a low walking speed [14, 15].

A particularly exciting future direction is to apply this approach to problems involving multiple control parameters on powered wearable devices. Though we only considered the efficacy of the algorithm under noisy measurement of metabolic cost using single parameter optimization, Bayesian optimization is generally applicable for multi-dimensional problems [28–32]. This method has been successfully applied to many robotic applications such as robot gait optimization [60, 61] and balancing recovery strategies under large disturbances [62]. In addition, another parameter selection application using a noisy physiological signal confirmed the sample efficiency of Bayesian optimization on a multi-dimensional problem during HIL optimization [33].

## 5 Conclusion

In this study, we evaluated the use of Bayesian optimization for optimizing human step frequency using noisy and slow metabolic cost signals. Our result demonstrate that Bayesian optimization rapidly identified near-optimal step frequencies, requiring only half of the time compared to an established gradient descent method. This significantly reduced the participants’ total energy expenditure, potentially expanding the inclusiveness of automatic parameter optimization studies. These results, combined with existing multidimensional applications of Bayesian optimization in robotics, make this class of algorithms a promising approach to achieving practical human-in-the-loop optimization of powered wearable devices.

## Supporting information

S1 File (XLSX)
